# Editorial for the Special Issue on Computational Quantum Physics and Chemistry of Nanomaterials

**DOI:** 10.3390/nano10122395

**Published:** 2020-11-30

**Authors:** Mojmír Šob

**Affiliations:** 1 Department of Chemistry, Faculty of Science, Masaryk University, Kotlářská 2, CZ-611 37 Brno, Czech Republic; sob@chemi.muni.cz or mojmir@ipm.cz or mojmir.sob@ceitec.muni.cz; 2 Institute of Physics of Materials, v.v.i., Czech Academy of Sciences, Žižkova 22, CZ-616 62 Brno, Czech Republic; 3 Central European Institute of Technology, CEITEC MU, Masaryk University, Kamenice 5, CZ-625 00 Brno, Czech Republic

Nanomaterials have become increasingly important both in basic research and in applications. Some properties may be understood only at the level of the quantum mechanical study of these materials. The purpose of this Special Issue is to advance our fundamental understanding of the structure and technologically important properties of nanomaterials with the help of computational quantum solid-state physics and chemistry. There is no doubt that quantum mechanical approaches are indispensable in comprehensive studies of nanomaterials and will be increasingly crucial in the future. Of course, this field is too extensive and too diverse to be described in a single volume. Nevertheless, this Special Issue provides at least a partial snapshot of the state of the art of computational quantum mechanical studies of nanomaterials and covers some recent advances and problems.

The scope of the articles included in this Special Issue is quite diverse, including adsorption of gas molecules on nanoclusters [1], domain structure of magnetically doped topological insulators [2], properties of dye-sensitized solar cells [3], energetics of silver decahedron nanoparticles [4], the effect of the size and shape on the surface energy of Au nanoparticles [5], critical size of ferroelectric SnTe low-dimensional nanostructures [6], the effect of vacancies on grain boundary segregation in ferromagnetic nickel [7], generalized stacking-fault energy in selected high-entropy alloys [8], phase transition of cesium lead halide perovskite nanocrystals [9], structural evolution of AlN nanoclusters [10] and the shock sensitivity of selected energetic materials [11].

In all these cases, application of the quantum methods was indispensable to determine how various features of atomic configuration of these materials are reflected in their properties and experimentally ascertained quantities. 

In summary, this Special Issue of *Nanomaterials* collects a series of original research articles providing new insight into the application of computational quantum physics and chemistry in research on nanomaterials. It illustrates the extension and diversity of the field and indicates some future directions. I am confident that this Special Issue will provide the reader with an overall view of the latest prospects in this fast evolving and cross-disciplinary field.
